# Retrospective study of adenovirus in autopsied pulmonary tissue of pediatric fatal pneumonia in South China

**DOI:** 10.1186/1471-2334-8-122

**Published:** 2008-09-21

**Authors:** Zhi-Ying Ou, Qi-Yi Zeng, Feng-Hua Wang, Hui-Min Xia, Jun-Peng Lu, Jian-Qing Xia, Si-Tang Gong, Li Deng, Jian-Tao Zhang, Rong Zhou

**Affiliations:** 1 Guangzhou Children's Hospital, Guangzhou Medical College, Guangzhou, 510120, Guangdong, PR China; 2 Department of Pediatrics, Nangfang Hospital, Southern Medical University, Guangzhou, 510301, Guangdong, PR China; 3 College of Veterinary Medicine, South China Agricultural University, Guangzhou, 510642, Guangdong, PR China

## Abstract

**Background:**

Adenovirus are the important pathogen of pediatric severe pneumonia. The aim of this study is to analyze the infection, subtype and distribution of adenovirus in autopsied pulmonary tissue of fatal pneumonia in infants and children, and the relationships between adenovirus infection and respiratory illness in South China.

**Methods:**

Nested PCR was performed on DNA extracted from autopsied lung tissue from patients who died of severe pneumonia, and the positive nested PCR products were cloned and sequenced. The adenovirus in autopsied pulmonary tissue was also analyzed by immunohistochemistry assay in a blind way.

**Results:**

In the 175 autopsied pulmonary tissues, the positive percentage of adenovirus was 9.14% (16/175) and 2.29% (4/175) detected with nested PCR and immunohistochemistry, respectively. There are three cases of adenovirus serotype 3, twelve cases of adenovirus serotype 4 and one case of serotype 41 determined by sequencing of the cloned positive nested PCR products.

**Conclusion:**

Adenovirus is an important cause of severe pneumonia, and these data suggest that adenovirus serotype 4 might be an important pathogen responsible for the fatal pneumonia in Guangzhou, South China.

## Background

Adenoviruses (AdVs) are non-enveloped, double-stranded DNA viruses that vary in size from 70 to 100 nm. They are the common pathogens of respiratory tract, gastrointestinal tract, urethral canal and eye characterized by self-limiting infection[1]. AdVs are endemic in children, and infections usually occur at young age (5 months to 6 years)[2,3]. AdVs can incite continuous and intensive infection with the utilization of a detective or compromised immune system sometimes resulting in lethality[4]. The clinical symptoms of AdVs infection are atypical and variable, including amygdalitis, conjunctivitis, pneumonia, gastroenteritis, hepatitis and hemorrhagic cystitis[2,3,5-9]. Local infections generalize in some cases and then have a mortality rate of up to 60%[2,3,5,7,8,10]. AdVs are the important pathogen of pediatric severe pneumonia, which can cause large-scale epidemics. For example, AdVs pneumonia was the most common pneumonia in Beijing and Shanghai area of China from the 1950s to 1960s characterized by complications, high mortality, but the incidence declined in the late 1980s with some diverging occasionally [11-13]. As reported from Zhang[14], viral pneumonia is mainly caused by respiratory syncytial virus (RSV) and AdVs in large cities, with an infection rate of 10%–15% respectively[15]. Adenovirus accounted for 32.2% of severe pneumonia as indicated from the report of Liu et al[16].

The clinical features of AdVs pneumonia in infants and children are emergency onset, violent background, and persistent ardent fever up to 40°C. Severe toxic symptoms all over the body occur in the early stages of infections, with weak consciousness, lethargy and cataphora. Respiratory symptoms include cough, asthma and anhelation. Cardiac and respiratory failures are common occurrences, as well as the fatality rates up to 20%–25%. The aim of this study is to analyze the AdVs infection associated with severe pediatric pneumonia and the relationships between AdVs infection and respiratory diseases. These data should provide information towards more accurate diagnosis and treatment of pediatric severe pneumonia.

## Methods

### Clinical samples

One hundred and seventy five cases of pediatric fatal pneumonia archived paraffin-embedded autopsied pulmonary tissues collected from July 1988 to January 2005 were analyzed in this study. All the samples were collected from Guangzhou Children's Hospital which included 114 male and 61 female specimens, aged one month to 10 years old, with an average age of 17.6 months. All the cases were pathologically diagnosed for interstitial or bronchial pneumonia in autopsia, among which 18 cases were pathologically diagnosised with AdVs pneumonia according to the cytopathology. The specimens were stored at room temperature until the viral genomic DNA was extracted and immunohistochemistry (IHC) was performed.

### Immunohistochemistry

Autopsied tissues were fixed in 10% buffered formalin for 24–48 hours, embedded in paraffin, and sectioned 4 μm thick onto Vectabond treated slides. Tissues were deparaffinized in two changes of xylene and re-hydrated from graded alcohols to distilled water. Tissues were quenched with 3% hydrogen peroxide for one hour. For the primary antibody, mouse-derived monoclonal antibodies against human AdVs hexon (Huayin, Guangzhou, China) were used at a 1:500 dilution and incubated for 1 hour at 37°C. Horseradish peroxidase (HRP) labeled secondary antibody included in the *MaxVision*™ HRP-Polymer anti-mouse/rabbit IHC kit (MaxVision, Fuzhou, China), was applied for 30 minutes at room temperature, followed by 2–6 minutes incubation at room temperature with diaminobenzidine (Dako Corp., CA) for color development. Slides were counterstained with hematoxylin (Harleco^®^), washed, and dehydrated with alcohol and xylene. Slides were mounted with a cover slip using a permanent mounting medium (Permount).

### DNA extraction

DNA was extracted from paraffin-embedded pulmonary tissue using DNeasy Kit for Purification of Total DNA from Animal Tissue (Qiagen, Germany). The procedures were as follows: 1) Five to eight pieces of 10 μm thick section of paraffin-embedded lung tissue were placed into 1200 μl xylene, vortexed vigorously and centrifuge at 14,000 rpm for 3 min at room temperature. 2) The supernatant was removed and 1200 μl ethanol (96%–100%) was added to the pellet to remove the residual xylene and mixed gently by vortex, the sample was centrifuged at 16,000 rpm for 5 min at room temperature and the supernatant removed. 3) Step 2 was repeated followed by continuation with the manufacturer's protocol: "Purification of Total DNA from Animal Tissues". Finally, DNA was eluted with 200 μl Buffer AE.

### Nested PCR conditions

Nested PCR on extracted DNA was performed with degenerate primers according to Allard et al[17], the primers are located in the hexon gene. Five microliters of DNA was amplified in 25 μl 1 × PCR buffer containing 200 μmol/L of each deoxynucleoside triphosphate (dNTP), 1.5 mmol/L of Mg^2+^, 1 U of *Taq *DNA polymerase (Promega, USA), and 0.2 μmol/L of each primer. The first round amplification was performed by using a T3 thermocycler (Biometra, Germany) under the following conditions: Initial denaturation at 95°C for 5 min, followed by 30 cycles of 95°C for 30 s, 50°C for 30 s, and 72°C for 30 s, and a final extension at 72°C for 7 min. The second round amplification was amplified with 3 μl of first round PCR product and was performed with the following conditions: Initial denaturation at 95°C for 5 min, followed by 35 cycles of 95°C for 30 s, 55°C for 30 s, and 72°C for 30 s, and a final extension at 72°C for 7 min. The PCR products were separated by electrophoresis through a 2% agarose gel and viewed under viltalight lamp.

### DNA cloning and sequencing

The second round PCR products were purified by phenol-chloroform (1:1) extraction, precipitated with ethanol, and dissolved in water. The 171 bp products were cloned into pMD 18-T Simple Vector (TaKaRa, Japan) according to the manufacturer's instruction. Cloned plasmids were sequenced in BioAsia Corporation (Shanghai, China).

### Blind Assay

Both nested PCR and IHC were carried out for all the samples. IHC results were confirmed by two pathologists. Positive results of nested PCR and IHC were repeated three times to confirm the results.

### Data analysis

All data were analysed by using SPSS for Windows version 11.0 (Chicago, IL, USA).

### Ethical aspects

Use of clinical samples in this research was authorised by the ethic committee of the Guangzhou Children's Hospital. The informed consents were signed with the guardians of the children studied. The study corresponded to the ethic committee standards of Guangzhou Children's Hospital.

## Results

### Immunohistochemistry

**Figure 1:**
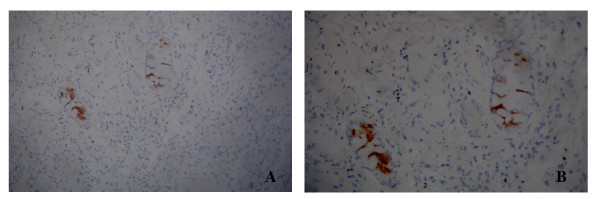
**Immunohistochemistry results of autopsied pulmonary tissue**. Labeling: A: 100 × (positive), B: 200 × (positive).

The representative positive results of AdVs infected pulmonary tissue are shown in Figure 1. The position of virus infection is distinct, locating in the cytoplasm of acinous cells in the mucilaginous glands under the tunica mucosa bronchiorum. Only 4 cases showed AdVs positive resulting in a positive rate of 2.29% (4/175).

### Nested PCR

**Figure 2:**
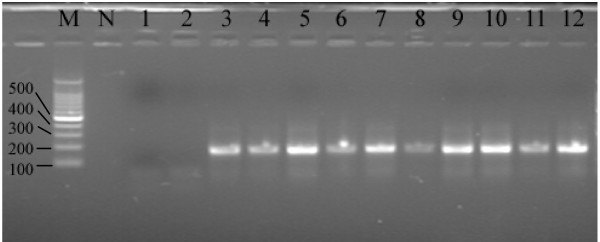
**Agarose gel electrophoresis analysis of nested PCR products in autopsied pulmonary tissues**. Labeling: M: DNA molecular marker, N: negative control, 1–2: negative cases, 3–12: positive cases.

DNA was extracted from 175 autopsied pulmonary tissues and nested PCR was performed with degenerate primers. The results for 12 of the 175 samples are shown in Figure 2. The presence of the 171 bp target band was seen in 16/175 samples. All the results are repeated three times with the same extraction DNA. Therefore, AdVs was detected in autopsied pulmonary tissue at a positive rate of 9.14%.

### DNA cloning and sequencing

The positive nested PCR products were purified, cloned and sequenced. The sequences acquired were subjected to BLAST analysis in Genbank. The results of this analysis were three cases (18.75%) of AdVs serotype 3, twelve cases (75%) of AdVs serotype 4 and one case (6.25%) of AdVs serotype 41 (Table 1).

**Table 1:** Distribution of adenovirus serotypes in autopsied pulmonary tissue of severe pneumonia

**AdV serotype**	**Cases**	**Percentage in AdV Positive Cases**
3	3	18.75%
4	12	75%
41	1	6.25%

### Comparison of pathological diagnosis, IHC and nested PCR

Nested PCR and IHC were blindly assayed for all the autopsied pulmonary tissues. Four cases were positive for both assays, 159 cases were negative for both assays, and 12 cases were positive in nested PCR but negative in IHC. There is no case in which the PCR results were negative and IHC results were positive (Table 2). Results of pathological diagnosis, nested PCR and IHC were also compared. There was only one case positive in three assays, 2 cases were positive in pathological diagnosis and nested PCR but negative in IHC, 15 cases were positive in pathological diagnosis but negative in nested PCR and IHC, 10 cases were negative in pathological diagnosis and IHC but positive in nested PCR, 3 cases were negative in pathological diagnosis but positive in nested PCR and IHC. The others were all negative in three assays (Table 3).

**Table 2:** Results of coincidence of nested PCR and IHC in AdV detection in autopsied pulmonary tissue of severe pneumonia

	**PCR Positive**	**PCR Negative**
IHC Positive	4 (2.29%)	0 (0%)
IHC Negative	12 (6.86%)	159 (90.86%)

**Table 3:** Results of comparison of pathological diagnosis, nested PCR and IHC in 175 cases of autopsied pulmonary tissue of severe pneumonia

**Pathologic diagnosis**	**Nested PCR**	**IHC**	**Number of Cases**
+	+	+	1
-	+	+	3
+	+	-	2
-	+	-	10
+	-	-	15
-	-	-	144

## Discussion

AdVs were the important agent of pediatric severe pneumonia, which can induce large-scale epidemics. AdVs infection was one of the common causes of pneumonia in Beijing and Shanghai area of China from the 1950s to 1960s, with more complications and high fatality rates. In the late 1980s, the incidence declined with only some diverging cases.

RSV and AdVs are the major agents of viral pneumonia in large cities, accounting for 10%–15% respectively[14]. AdVs accounts for 32.2% of severe pneumonia according to Liu et al[16]. The results of virus separation in cell culture in our hospital from 1975 to 1985 showed that the years 1976, 1979, and 1980 were the highly epidemic years (data not shown). Results from ELISA and virus cultures for respiratory disease from January 2003 to December 2005 in our hospital showed the AdVs positive rate was 8.5% (70/824) (data not shown). Our results show that AdVs infection in autopsied severe pneumonia pulmonary tissues was 9.14%. Taken together, these observations indicate that AdVs is still a major agent of severe pneumonia and it plays an important role in lethal severe pneumonia in Guangzhou area. Therefore, we must strengthen the surveillance for AdVs and national recommendations for AdVs vaccine development in future.

AdVs are responsible for much of the public incidence of pneumonia. The 51 different serotypes of human AdVs are classified into six subgenera (subgenera A to F) on the basis of erythrocyte coagulation characteristics, oncogenicity and DNA sequence[2]. The clinical pathogenicity differs among different adenovirus serotypes. The AdVs types within a subgenus are similar in their tropisms, pathogenicity, and tendencies to cause a latent infection and epidemics[17,18]. Overall frequency of AdVs as the cause of nonbacterial pneumonia in children is less than that of RSV and parainfluenza virus type 3, but an alarming number of fatal illnesses have been noted. Severe and fatal illnesses in infants and children have been noted in association with AdVs types 1, 2, 3, 4, 5, 7a, 7h, 7i, 8, 19, 21, 35, and the intermediate strain 21/H21+35[19]. Respiratory illness is mainly caused by AdVs serotype 3, 4, 7, 14, 21, and to a less extent by serotypes 1, 2, 5 and 6[6,18]. Pediatric pneumonia is mainly caused by AdVs serotypes 1, 2, 3 and 7, whereas serotypes 4 and 7 are mainly responsible for adult pneumonia. According to several reports, serotypes 3, 4, 7 and 21 usually cause large scale respiratory illnesses and fulminate prevalence events, causing respiratory illness such as severe lethal pneumonia and pharyngoconjunctival fever [20,21]. Our data identified 12 cases of AdVs serotype 4 from 175 autopsied pulmonary tissues, suggesting that AdVs serotype 4, which accounts for 75% in the positive cases, might be an important serotype responsible for pediatric fatal pneumonia in the Guangzhou area which locates in South China, and therefore it is important that we pay close attention to the detection of AdVs serotype 4 in pediatric severe pneumonia.

Our results from nested PCR and IHC are in concordance. Twelve PCR positive cases are negative in IHC, suggesting that nested PCR is a more sensitive method for the detection of AdVs and also reflecting the limitation of IHC in pathogen detecting. Results of pathological diagnosis, AdVs nested PCR and IHC were compared. The cases were all negative or positive in the three assays, or positive in pathological diagnosis, nested PCR but negative in IHC representing the coincidental cases with a concordance percentage of 84% (147/175), while others were not as concordant (Table 3). The unmatched results in the three methods indicated that PCR is a more sensitive assay than IHC. Pathological diagnosis of cell morphologic is to a large extent dependent on observation under the microscope with the naked eyes, therefore, the standards are subjective in some degree, which can result in poor specificity, high misdiagnosis or missed rate. Thus, pathological diagnosis has limitations compared with the sensitive, specific and reproducibility of nested PCR assay at the molecular level. As for the archived paraffin-embedded autopsied pulmonary tissues stored from 1988 to 2005 for PCR, the 16 positive samples distribute from 1994 to 2005 but none before 1993. The 15 cases of positive pathological diagnosis without PCR positivity (Table 3), it is possible that these cases or a number of them were older than 15 years, i.e. originating from before 1993, Perhaps the viral DNA in the older samples stored at room temperature more than 15 years degraded seriously and the materials is too old to be assayed by PCR. Concerning severe pneumonia in the clinic, we must strengthen the pathogen detection based on PCR technology, such as fluorescent quantitative PCR, nested fluorescent quantitative PCR, and nested PCR in order to provide more accurate and reliable molecular diagnostics results for pathogen identification underlying the disease and thus avoid the abuse of antibiotics.

## Conclusion

Adenovirus is an important cause of severe pneumonia, and these data suggest that adenovirus serotype 4 might be an important pathogen responsible for the fatal pneumonia in Guangzhou, South China.

## Competing interests

The authors declare that they have no competing interests.

## Authors' contributions

ZYO has been involved in designing the study and drafting the manuscript or revising it critically for important intellectual content. QYZ has made substantial contributions to conception and design. FHW carried out the IHC experiment as a pathologist. HMX participated in the sequence alignment and polished the manuscript. JPL carried out the nested PCR. JQX participated in the IHC experiment. STG participated in the design of the study. LD participated in its design and performed the statistical analysis. JTZ carried out the IHC experiment as a pathologist. RZ has given final approval of the version to be published. All authors read and approved the final manuscript.

## Pre-publication history

The pre-publication history for this paper can be accessed here:


